# Supplementary figures and images for: Differential chromatin accessibility landscape of gain-of-function mutant p53 tumours
Source: BMC Cancer. 2021 Jun 5;21:669. doi: 10.1186/s12885-021-08362-x (PMC8180165; doi:10.1186/s12885-021-08362-x)

Figure S1

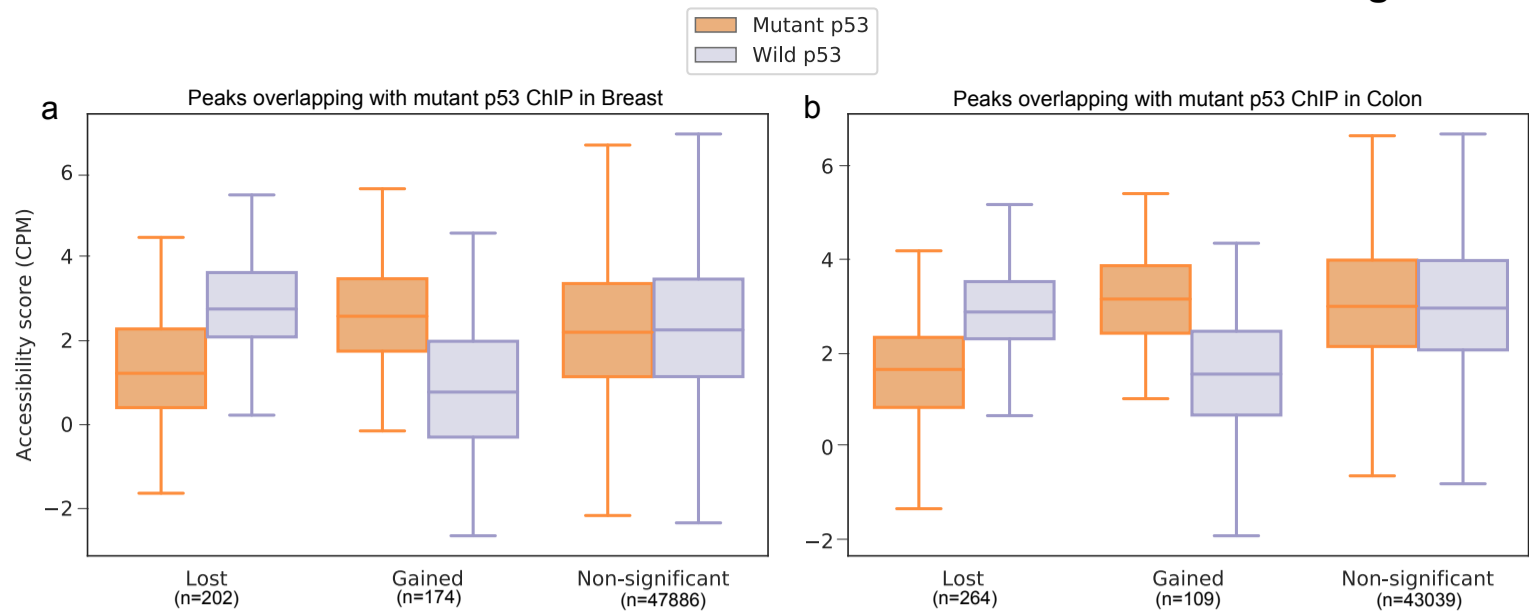

Figure S2

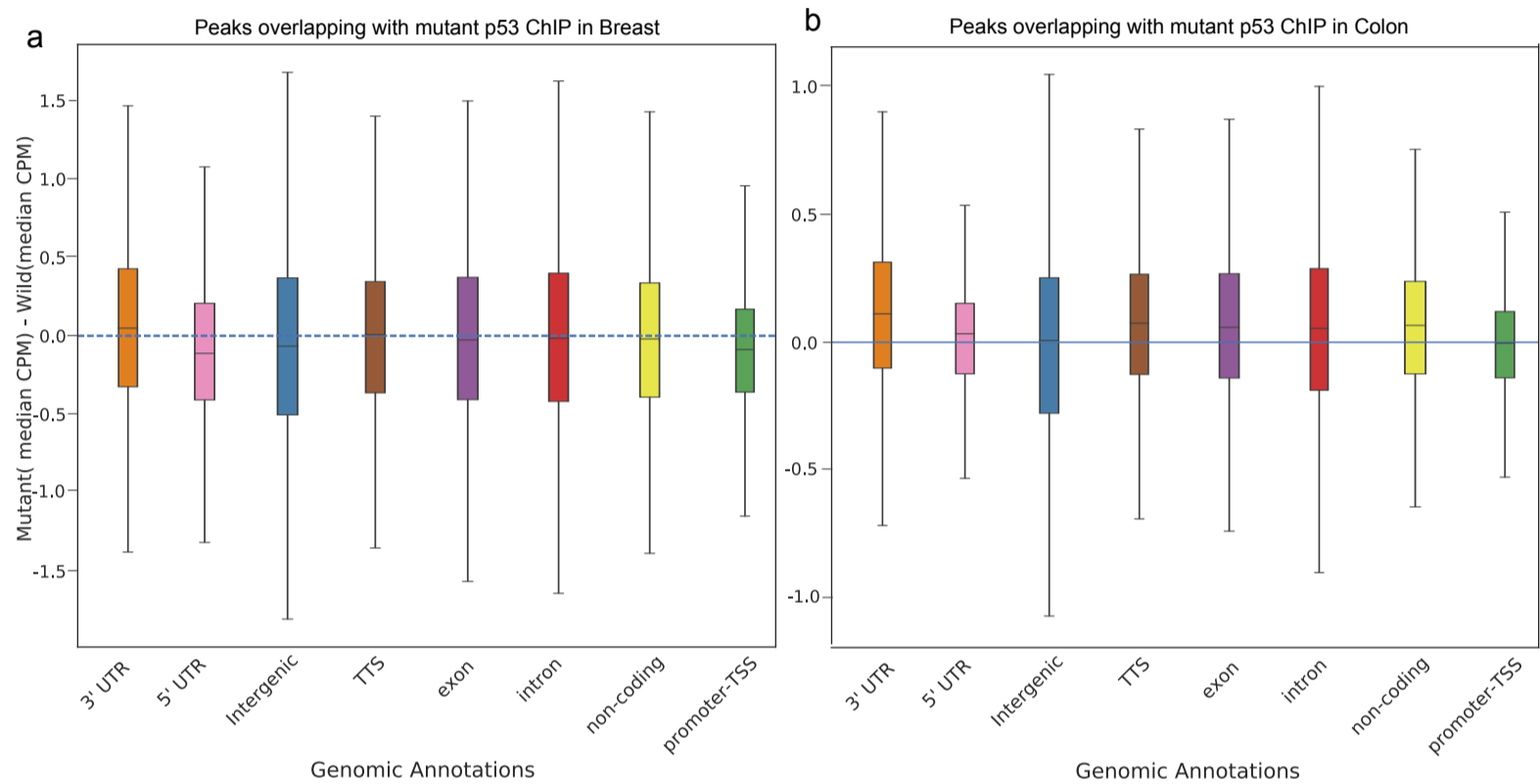

Figure S3

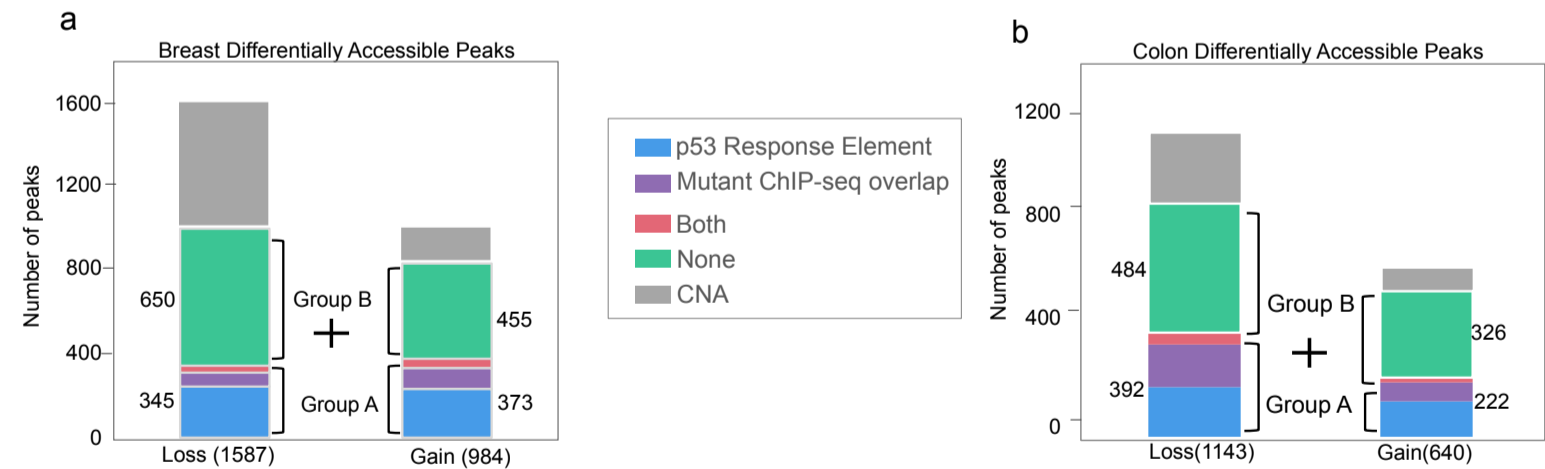

Figure S4

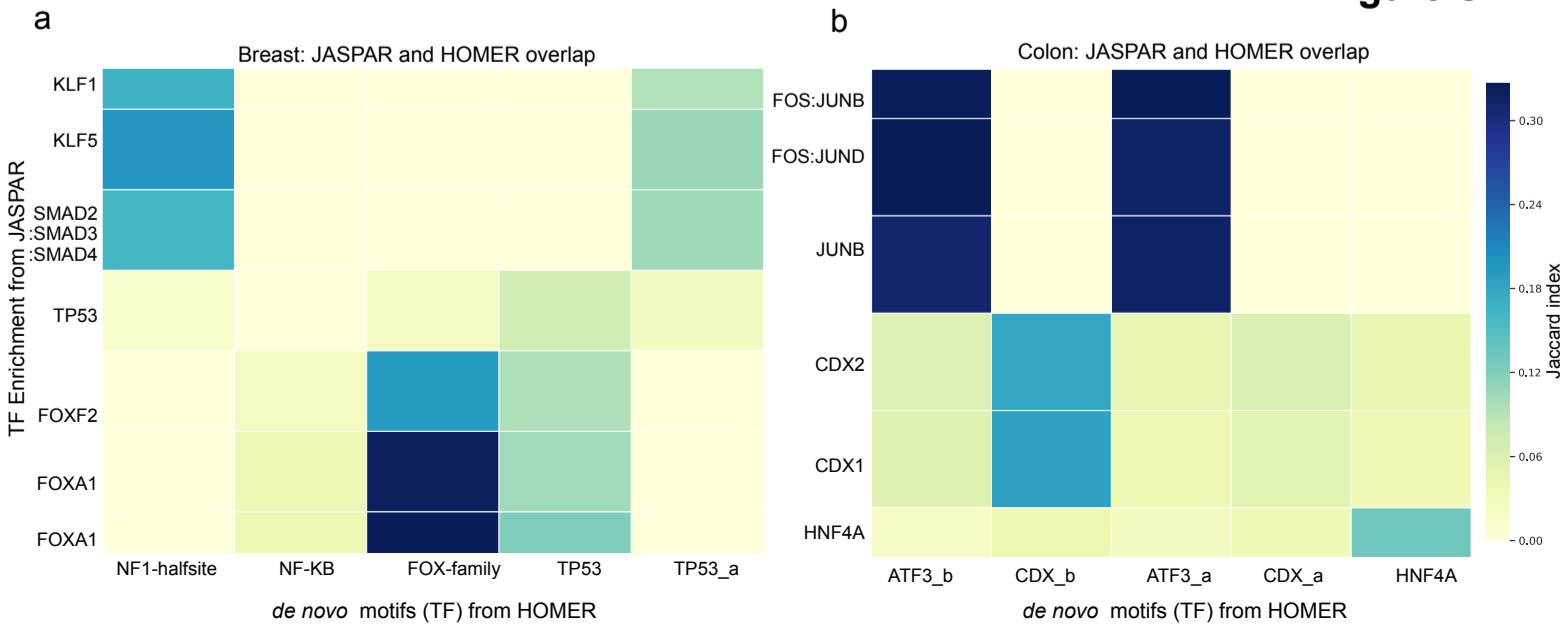

Supplement: Supplementary file 2 — Additional file 2: Figure S1. The box-plot shows the distribution of accessibility scores (CPM) in wild-type and mutant p53 samples for peaks that overlapped the mutant p53 binding sites (inferred from ChIP-seq data). The peaks were split into three groups: non-significant, significant gained and significant lost, as shown on the x-axis. Figure S2. The box-plot shows the distribution of the difference between the accessibility scores (CPM) between wild-type and mutant p53 tumours in peaks (both significant and non-significant) that overlapped the mutant p53 binding sites (inferred from ChIP-seq data). The peaks were split based on the genomics annotations as shown on the x-axis. Figure S3. The significant gained and lost accessible peaks identified in breast and colon cancer have been categorized into those that overlapped regions with potentially copy number alterations, those that predicted to have p53 response element, overlap with mutant p53 binding sites (inferred from ChIP-seq data), both or none. Figure S4. The heatmap represents the overlap of differentially accessible peaks predicted with TF motifs from JASPAR and de novo motif analysis from HOMER. The colour scale represents the Jaccard index score. The higher the value the higher the overlap between the genomic regions. [file 12885_2021_8362_MOESM2_ESM.pdf]
