# Supplementary material for: Differential chromatin accessibility landscape of gain-of-function mutant p53 tumours
Source: BMC Cancer. 2021 Jun 5;21:669. doi: 10.1186/s12885-021-08362-x (PMC8180165; doi:10.1186/s12885-021-08362-x)
Supplement: Supplementary file 3 — Additional file 3. de novo motifs search result from HOMER analysis in the differentially accessible peaks of breast and colon cancers. [file 12885_2021_8362_MOESM3_ESM.pdf]

Additional File 3: de novo motifs result from HOMER analysis of group A and B differentially accessible peaks.

Breast Group A Gain (target= 373; background= 49288)

Rank	Motif	P-value	log P-pvalue	% of Targets	% of Background	STD(Bg STD)	Best Match
1	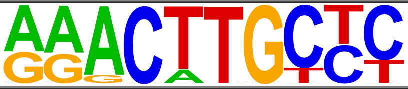	1e-36	-8.293e+01	37.27%	12.36%	140.6bp (155.7bp)	P53/Jaspar
2	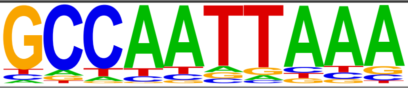	1e-13	-3.108e+01	27.08%	12.50%	147.8bp (152.7bp)	LHX2/Jaspar

Breast Group A Loss (target= 345; background= 49264)

Rank	Motif	P-value	log P-pvalue	% of Targets	% of Background	STD(Bg STD)	Best Match
1	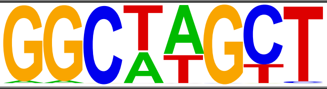	1e-40	-9.21e+01	62.90%	30.11%	130.7bp (154.7bp)	P53/Jaspar
2	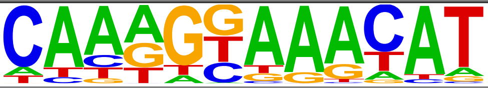	1e-15	-3.485e+01	17.10%	5.15%	138.3bp (148.4bp)	FOXA1

Breast Group B Gain (target= 455; background= 49276)

Rank	Motif	P-value	log P-pvalue	% of Targets	% of Background	STD(Bg STD)	Best Match
1	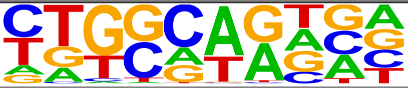	1e-23	-5.419e+01	44.40%	22.84%	119.9bp (157.3bp)	NF1-halfsite
2	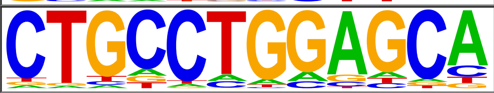	1e-12	-2.943e+01	16.70%	6.64%	130.9bp (157.6bp)	TFAP2B

Breast Group B Loss (target= 650; background= 49101)

Rank	Motif	P-value	log P-pvalue	% of Targets	% of Background	STD(Bg STD)	Best Match
1	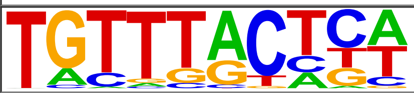	1e-71	-1.634e+02	42.31%	19.43%	111.4bp (148.7bp)	FOX_Family
2	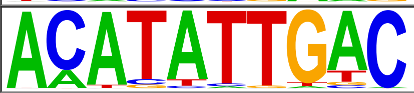	1e-27	-6.216e+01	10.31%	2.73%	121.0bp (142.5bp)	Hnf6b
3	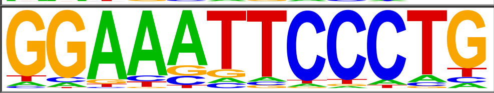	1e-27	-6.216e+01	6.92%	1.75%	114.8bp (144.4bp)	NFkB-p65-Rel
4	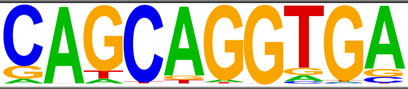	1e-12	-2.947e+01	8.15%	2.51%	130.2bp (154.5bp)	Zic1::Zic2

Colon Group A Gain (target= 222; background= 47919)

Rank	Motif	P-value	log P-pvalue	% of Targets	% of Background	STD(Bg STD)	Best Match
1	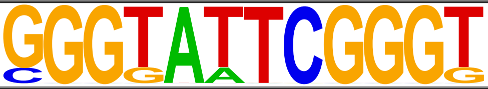	1e-13	-3.170e+01	2.25%	0.00%	77.2bp (67.9bp)	GCM2
2	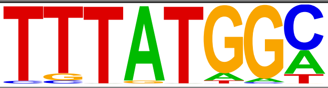	1e-13	-2.999e+01	36.04%	15.67%	128.9bp (153.0bp)	CDX_Family
3	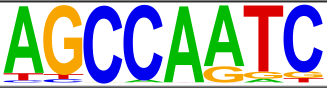	1e-12	-2.905e+01	33.33%	13.99%	121.0bp (151.4bp)	POL004.1/Jaspar
4	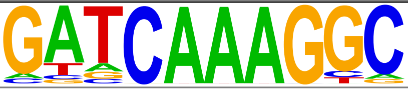	1e-12	-2.797e+01	9.91%	1.35%	110.0bp (149.7bp)	PB0040.1/Jaspar

Colon Group A Loss (target= 392; background= 49626)

Rank	Motif	P-value	log P-pvalue	% of Targets	% of Background	STD(Bg STD)	Best Match
1	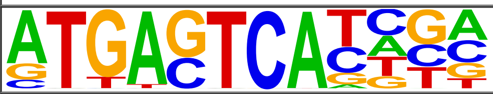	1e-217	-4.999e+02	44.16%	4.86%	75.9bp (152.0bp)	ATF3
2	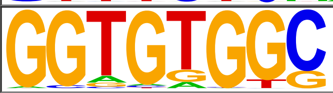	1e-19	-4.555e+01	30.46%	12.67%	108.8bp (152.3bp)	Klf4
3	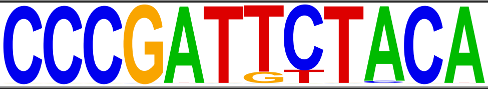	1e-16	-3.749e+01	2.79%	0.04%	179.5bp (152.7bp)	PB0132.1/Jaspar
4	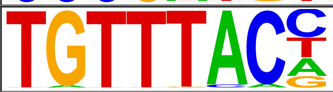	1e-15	-3.543e+01	17.51%	5.83%	114.1bp (151.7bp)	Foxo3
5	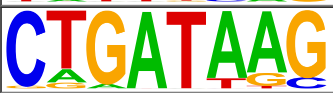	1e-14	-3.348e+01	18.02%	6.37%	119.8bp (154.4bp)	PB0022.1/Jaspar

Colon Group B Gain (target= 326; background= 49101)

Rank	Motif	P-value	log P-pvalue	% of Targets	% of Background	STD(Bg STD)	Best Match
1	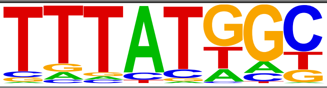	1e-33	-7.59e+01	38.70%	14.33%	113.2bp (148.6bp)	CDX1
2	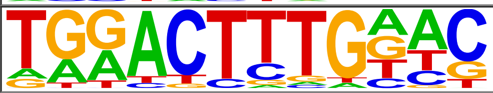	1e-27	-6.353e+01	21.98%	4.57%	103.4bp (149.3bp)	HNF4A

Colon Group B Loss (target= 484; background= 49414)

Rank	Motif	P-value	log P-pvalue	% of Targets	% of Background	STD(Bg STD)	Best Match
1	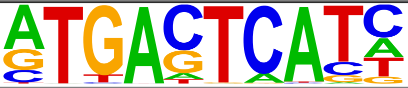	1e-120	-2.762e+02	43.06%	8.45%	75.7bp (151.5bp)	ATF3
2	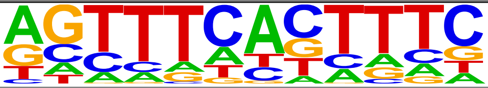	1e-29	-6.697e+01	23.40%	7.04%	118.8bp (148.1bp)	ISRE(IRF)
3	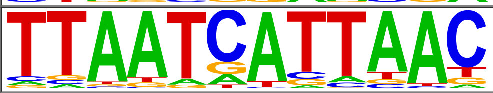	1e-22	-5.197e+01	11.18%	2.07%	111.3bp (154.7bp)	HNF1b
4	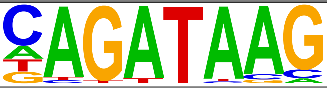	1e-17	-4.030e+01	49.48%	30.56%	136.9bp (149.5bp)	GATA2
5	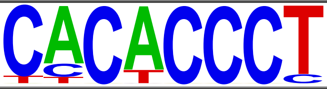	1e-15	-3.663e+01	27.12%	13.01%	117.8bp (149.4bp)	Klf1
6	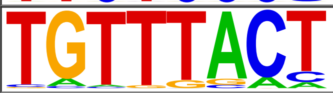	1e-14	-3.335e+01	32.51%	17.72%	118.4bp (149.9bp)	FOXP1
